# The landscape of driver mutations in cutaneous squamous cell carcinoma

**DOI:** 10.1038/s41525-021-00226-4

**Published:** 2021-07-16

**Authors:** Darwin Chang, A. Hunter Shain

**Affiliations:** 1 University of California San Francisco, Department of Dermatology, San Francisco, CA USA; 2 University of California San Francisco, Helen Diller Family Comprehensive Cancer Center, San Francisco, CA USA

**Keywords:** Cancer genomics, Squamous cell carcinoma

## Abstract

Cutaneous squamous cell carcinoma is a form of skin cancer originating from keratinocytes in the skin. It is the second most common type of cancer and is responsible for an estimated 8000 deaths per year in the United States. Compared to other cancer subtypes with similar incidences and death tolls, our understanding of the somatic mutations driving cutaneous squamous cell carcinoma is limited. The main challenge is that these tumors have high mutation burdens, primarily a consequence of UV-radiation-induced DNA damage from sunlight, making it difficult to distinguish driver mutations from passenger mutations. We overcame this challenge by performing a meta-analysis of publicly available sequencing data covering 105 tumors from 10 different studies. Moreover, we eliminated tumors with issues, such as low neoplastic cell content, and from the tumors that passed quality control, we utilized multiple strategies to reveal genes under selection. In total, we nominated 30 cancer genes. Among the more novel genes, mutations frequently affected *EP300*, *PBRM1*, *USP28*, and *CHUK*. Collectively, mutations in the NOTCH and p53 pathways were ubiquitous, and to a lesser extent, mutations affected genes in the Hippo pathway, genes in the Ras/MAPK/PI3K pathway, genes critical for cell-cycle checkpoint control, and genes encoding chromatin remodeling factors. Taken together, our study provides a catalog of driver genes in cutaneous squamous cell carcinoma, offering points of therapeutic intervention and insights into the biology of cutaneous squamous cell carcinoma.

## Introduction

Over the past decade, large-scale DNA-sequencing studies have profiled a wide range of different cancers^1,2^. These studies have revealed candidate genes for targeted therapy and genetically distinct subtypes of cancer—information that has changed the way in which many cancers are treated. Moreover, at a basic science level, these studies have revealed fundamental insights into the biology of these cancers, often forming the basis of downstream hypothesis-driven work.

Given these achievements, there has been momentum to genomically profile the rarest of cancer subtypes^1,2^, yet cutaneous squamous cell carcinoma, the second most common form of cancer in the United States^3^, has largely been overlooked. Thirty-four cancer subtypes were included in The Cancer Genome Atlas program (TCGA)—a comprehensive effort to catalog the driver genes in cancer—but regrettably, cutaneous squamous cell carcinoma was left out. Several individual laboratories have sequenced the exomes or genomes of cutaneous squamous cell carcinomas, examples of which are here^4–13^, but the small size of each study and difficulties in interpreting the high mutational loads in cutaneous squamous cell carcinomas have precluded the research community from settling upon a consensus set of driver genes. Indeed, power calculations suggest that the largest exome study to date can only recognize genes under positive selection in cutaneous squamous cell carcinoma that are mutated in ~50% or more of tumors^14,15^.

One reason why large-scale sequencing consortiums have overlooked cutaneous squamous cell carcinomas is because cutaneous squamous cell carcinomas are thought of as non-life-threatening tumors; however, this reputation is misleading. Most cutaneous squamous cell carcinomas are caught at an early stage, reducing their mortality, but 8000 people per year still die from this disease in the United States^3,16–18^. To put this death toll in perspective, it is on par with that of melanoma^19^, for which nearly 1000 tumors have been sequenced, to date, at exome or genome resolution^20^.

A better understanding of the genetic drivers of cutaneous squamous cell carcinoma promises to improve treatment strategies. The current standard of care is for patients to receive immune-checkpoint blockade therapies, but roughly half do not respond and the responses are not always durable^21^. In addition, the risk of cutaneous squamous cell carcinoma is nearly 100-fold higher in immunosuppressed patients, such as organ transplant recipients, who are typically not eligible to receive immunotherapies^22^. Establishing the driver mutations in cutaneous squamous cell carcinoma promises to reveal new points for therapeutic intervention in this deadly tumor subtype. Towards this goal, we performed a meta-analysis of publicly available exome-sequencing data from cutaneous squamous cell carcinomas.

## Results

### Assembling a cohort of cutaneous squamous cell carcinomas

We performed a literature search to identify whole-exome or whole-genome sequencing studies of cutaneous squamous cell carcinoma in which raw sequencing data were made publicly available. In total, we identified 105 tumors spanning 10 studies (Table 1 and Supplementary Data 1)^4–13^. We assessed the quality of sequencing data and removed 17 tumors from subsequent analyses (see “Methods” for exclusion criteria). The remaining 88 tumors were retained, though we accounted for our ability to call somatic mutations in each tumor before comparing them.

**Table 1:** Summary of exome or genome sequencing studies analyzed in this meta-analysis.

Studies	Sample size
Durinck, S. et al., *Cancer Discov*. (2011)^9^	8
Wang, N. J. et al., *PNAS* (2011)^11^	4
South, A. P. et al., *JID* (2014)^10^	20
Zheng, C. L. et al., *Cell Rep.* (2014)^12^	4
Cammareri, P. et al., *Nat. Commun*. (2016)^8^	10
Chitsazzadeh, V. et al., *Nat. Commun.* (2016)^6^	7
Yilmaz, A. S. et al., *Cancer* (2017)^4^	6
Cho, R. J. et al., *Sci. Transl. Med*. (2018)^5^	27
Inman, G. J. et al., *Nat. Commun*. (2018)^7^	10
Ji, A. L. et al., *Cell* (2020)^13^	9
Total	105

The number of tumors, listed here, corresponds to unique tumors, whose data were made publicly available, and thus may not match the reported size from the original studies.

The main issues affecting our ability to detect somatic mutations in each tumor were the neoplastic cell content, the mean sequencing coverage, and/or the variability in sequencing coverage. We bioinformatically quantified tumor cellularities, and they ranged from 12% to 99%. The mean sequencing coverages ranged from 12.4× to 498×. Finally, some tumors had high sequencing coverages, on average, but extreme variability in coverage, primarily linked to the GC content of their targets (see Supplementary Fig. 1a for an example). To account for each of these potential issues, we used the Footprints software^23^ to count the exact number of basepairs in each sample with sufficient sequencing coverage to make a mutation call. For the average sample, we could detect mutations at 91.2% of target bases, though this ranged from 52.3% to nearly 100% (Supplementary Fig. 1b).

Establishing the extent to which we could detect mutations in each tumor allowed us to accurately calculate mutation burdens, irrespective of technical variables that are known to distort these measurements^24^. Moreover, the performance of cancer gene discovery tools deteriorates when there are large portions of the exome for which a mutation call cannot be made, and we were able to exclude problematic tumors from these analyses (Supplementary Fig. 1b). Altogether, we improved the caliber of candidate cancer genes by aggregating a large cohort of tumors, reanalyzing the raw sequencing data, and applying rigorous quality control measures for sample inclusion.

### Subtypes of cutaneous squamous cell carcinoma

**Fig. 1:**
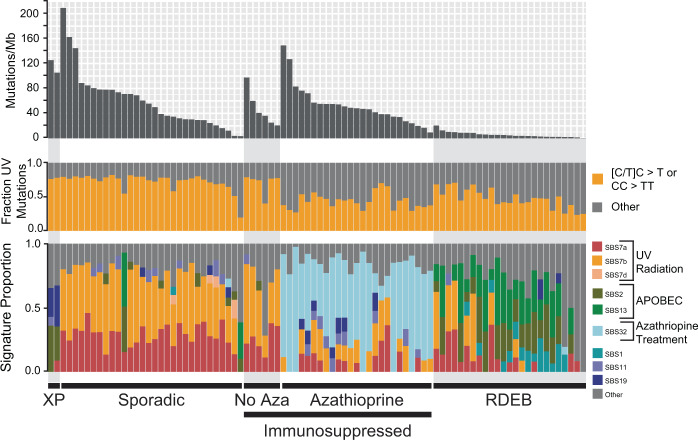
Subtypes of cutaneous squamous cell carcinoma. Each vertical bar corresponds to a single tumor. Top track: The mutation burden (mutations per megabase) of each tumor. Middle track: The fraction of mutations within each tumor matching the canonical UV radiation signature. Bottom track: The fraction of mutations attributable to established mutational signatures within each tumor. The proposed etiology of select signatures is indicated. XP = tumors from patients with xeroderma pigmentosum, Sporadic = tumors from patients with no known comorbidities, immunosuppressed: tumors from immunosuppressed patients—this group is further stratified by the usage or absence of azathioprine as an immunosuppressive drug, RDEB = tumors from patients with recessive dystrophic epidermolysis bullosa.

We calculated the mutation burden and the proportion of each tumor’s mutations that were attributable to established mutational signatures (Fig. 1), revealing five distinct subtypes of cutaneous squamous cell carcinoma among the tumors analyzed in this study.

First, two cutaneous squamous cell carcinomas came from patients with xeroderma pigmentosum—a rare hereditary disorder characterized by extreme sensitivity to UV radiation and caused by germline mutations in genes involved in nucleotide excision repair^25^. As expected, these two tumors had high mutation burdens with a high frequency of cytosine to thymine transitions at the 3′ basepairs of consecutive pyrimidines (the classic mutation that arises from UV radiation^26^). Interestingly, they did not have a high proportion of “signature 7” mutations (a mutational signature extracted from pan-cancer analyses and attributed to UV radiation^27,28^). The absence of signature 7 was due to differences in the trinucleotide contexts of mutations arising in wild type versus mutant-*XPC* tumors (Supplementary Fig. 2a) and illustrates how the repertoire of known mutational signatures remains incomplete.

Second, there were cutaneous squamous cell carcinomas that arose sporadically in patients with no known comorbidities. These cutaneous squamous cell carcinomas had high mutation burdens, and the majority of mutations were attributable to UV radiation (Fig. 1). The most common mutations in these tumors overlapped with mutations recently shown to be enriched in sun-exposed normal skin (Supplementary Fig. 2b), further linking UV radiation to their formation^29^.

Furthermore, there were two distinct types of cutaneous squamous cell carcinomas arising from immunosuppressed patients, who were primarily organ-transplant recipients. As previously reported^7^, cutaneous squamous cell carcinomas from patients treated with Azathioprine (as a means to prevent transplant rejection) had high mutation burdens with high proportions of signature 32 (Fig. 1). Azathioprine increases the risk of cutaneous squamous cell carcinoma beyond the risk conferred by other immunosuppressive agents^30^ because Azathioprine is both immunosuppressive and a potent mutagen^31,32^. Patients on other immunosuppressive drug regimens had comparably lower mutation burdens, primarily attributable to UV radiation (Fig. 1).

Finally, there were cutaneous squamous cell carcinomas from patients with recessive dystrophic epidermolysis bullosa (RDEB), a rare hereditary disorder characterized by chronic blistering in the skin and caused by germline mutations in collagen VII (*COL7A1*). As previously noted^5^, these tumors had relatively low mutation burdens, primarily from APOBEC-mediated mutagenesis (Fig. 1).

### Nomination of driver mutations in cutaneous squamous cell carcinoma

**Fig. 2:**
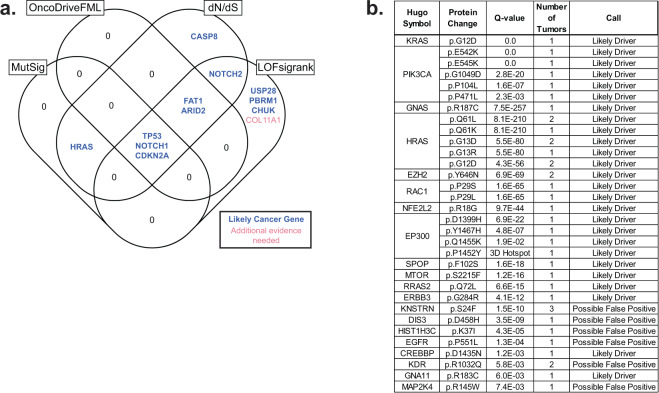
Nomination of cancer genes in cutaneous squamous cell carcinoma. **a** A Venn diagram depicting nominated cancer genes from four separate cancer gene discovery programs, each designed to identify genes under positive selection in cancer. The set of candidate genes were further curated, as described, to nominate candidates for which additional evidence is warranted (red text) or not (blue text). **b** A list of mutations in our study that overlap mutations in the cancerhotspots.org database. Mutations are grouped by gene and ordered by their *q* values (lowest to highest). These mutations were also curated, as described, to nominate candidates for which additional evidence is warranted (Possible False Positive) or not (Likely Driver).

Genes under positive selection in cancer are distinguished by having significantly more mutations than the background mutation rate at that locus would predict^33^. We utilized four cancer gene discovery tools to reveal such genes: MutSig^14^, dN/dS^34^, LOFsigrank^35^, and OncodriveFML^36^. Collectively, these tools nominated 12 genes total, including a subset of 7 genes by at least 2 tools (Fig. 2 and Supplementary Data 2).

While these tools nominated a relatively small number of genes, the number of genes nominated is in line with our statistical power to detect cancer genes in this study^15^. Specifically, the genes nominated by these tools tended to be mutated in approximately 15% or more of tumors. To nominate driver mutations that were too infrequent to show evidence of positive selection in this dataset alone, we searched for overlapping mutations in the cancerhotspots.org database (Fig. 2b). This database contains mutations identified from pan-cancer analyses that cluster within genes^37^—a common pattern for gain-of-function mutations. We reasoned that if a mutation shows evidence of positive selection from pan-cancer analyses, and the exact same mutation was present in our dataset, then it deserves consideration as a driver of cutaneous squamous cell carcinoma.

Finally, we nominated genes with focal copy number alterations. Focal, homozygous deletions affected the *CDKN2A* and *PTEN* tumor suppressor genes (Supplementary Fig. 3). A focal, heterozygous deletion affected *AJUBA* in one tumor, and in the same tumor there was a point mutation affecting the other allele (Supplementary Fig. 3b). Focal amplifications affected: *CCND1*, *MDM2*, *YAP1*, and *RAP1B* (Supplementary Fig. 4). *RAP1B* is a ras-related-protein, and the tumor with amplification of *RAP1B* also had a point mutation affecting the amplified allele. This point mutation was analogous to mutations known to activate other Ras genes (Supplementary Fig. 4e, f).

### Removal of false positive driver mutations

The earliest generation of algorithms to discover cancer genes assumed a background mutation rate that is uniform across the genome (an assumption that is not true^38^), resulting in the nomination of hundreds of candidate genes in high mutation burden cancers, most of which were large and poorly expressed^39,40^. The algorithms, utilized here, have improved background mutation rate models, but the determinants of the mutation rate across the genome are complex and remain incompletely understood^38^, leaving open the possibility of false positives. We curated the nominated genes, as described below, to root out unlikely cancer genes, though we acknowledge that these are ultimately judgment calls.

We concluded that *COL11A1* (Fig. 2a) requires additional evidence to be considered a driver gene. *COL11A1* has a borderline significant *q*-value (Supplementary Data 2). One reason why it was nominated was because it had a high number of splice-site mutations; however, it has an unusual gene structure with many small exons, increasing the probability that these mutations could have occurred by random chance. Moreover, *COL11A1* is poorly expressed in the keratinocyte lineage (Supplementary Fig. 5a, see Supplementary Note in the methods for more details on why we removed this gene from consideration). We also determined that *DIS3*, *HIST1H3C*, *KDR*, and *MAP2K4* (Fig. 2b) need additional evidence to be considered driver genes. The hotspot mutations affecting these genes had *q*-values that were low in comparison to others in the cancerhotspots.org database, and OncoKb, a precision oncology consortium at Memorial Sloan Kettering Cancer Center^41^, classifies these hotspot mutations as unlikely to be oncogenic.

Finally, we do not believe there is sufficient evidence to classify the hotspot mutation affecting *KNSTRN* as oncogenic. The mutation is annotated as coding, but this appears to be based on an erroneous gene model. From RNA-sequencing data of normal skin and cutaneous squamous cell carcinoma, expression of *KNSTRN* begins downstream of the mutation site (Supplementary Fig. 5c), implying that the mutation affects the promoter of *KNSTRN*. Promoter mutations are ubiquitous in sun-exposed cancers^42,43^ because transcription factors at the promoter can bend DNA in ways that render their binding elements vulnerable to mutagenesis by UV radiation^44^. These types of annotation errors are not uncommon—many hotspot mutations in melanoma, which were initially thought to be coding mutations, were subsequently revealed to be promoter mutations after further studies^20,35,45^. There is a study suggesting *KNSTRN*^*S24F*^ is oncogenic^46^, but the supporting evidence presumes the mutation is coding.

### Novel candidates in cutaneous squamous cell carcinoma

**Fig. 3:**
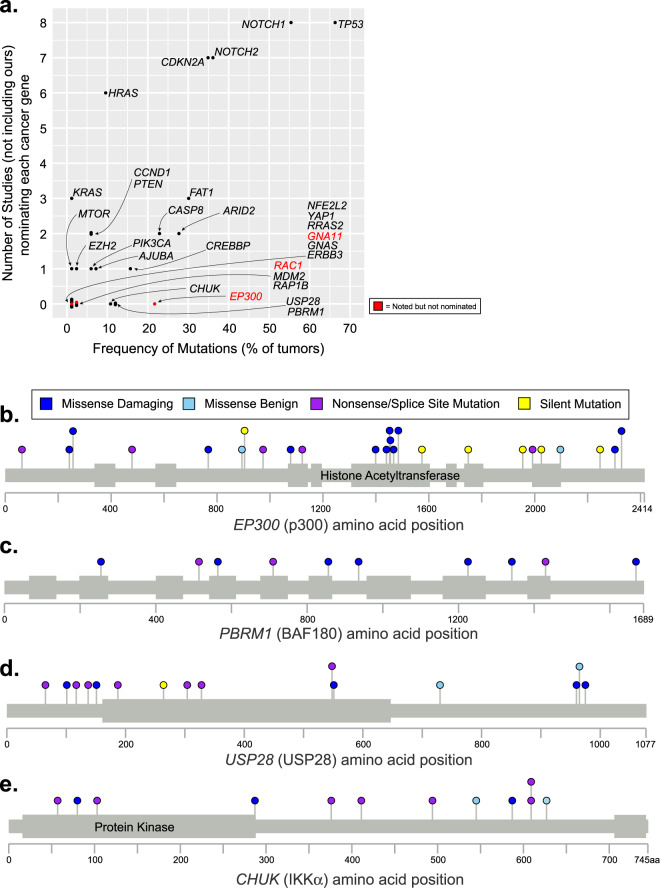
Candidate cancer genes in cutaneous squamous cell carcinoma. **a** The genes nominated in our meta-analysis are stratified by their mutation frequency (*x*-axis) and how often they were nominated in eight previous studies (*y*-axis) that cataloged drivers of cutaneous squamous cell carcinoma. We further inspected the genes nominated by us, but not others (those with a value of 0 on the *y*-axis). Some were borderline significant in other studies, which we designate in red text (see “Methods” for more details on the difference between “nominated” and “noted” genes). **b–e** *EP300*, *PBRM1*, *USP28*, and *CHUK* were mutated in greater than 10% of tumors but not nominated by other studies. Lollipop diagrams portray the spectrum of mutations in each of these four genes in panels.

We aggregated the genes nominated by the cancer gene discovery algorithms (Fig. 2a), the hotspot mutation analyses (Fig. 2b), and the copy number analyses (Supplementary Figs. 3 and 4). Next, we compared these genes, nominated in our meta-analysis, to those from eight other studies that have proposed cancer genes in cutaneous squamous cell carcinoma^5–10,47,48^ (Fig. 3). *TP53*, *NOTCH1*, *NOTCH2*, *CDKN2A*, and *HRAS* were proposed by a majority of the other studies and also nominated by us. Moreover, *FAT1*, *ARID2*, *CASP8*, *CREBBP*, *AJUBA*, *PTEN*, *PIK3CA*, *EZH2*, *KRAS*, *CCND1*, and *MTOR* were nominated in 1–3 studies each as well as by us, lending credibility to their pathogenic roles in cutaneous squamous cell carcinoma.

We nominated 13 genes that were not nominated in the other studies. Many of these genes harbored hotspot mutations that occurred relatively infrequently (see Fig. 2b for the full list), likely explaining why they were not noted in other analyses. However, four genes were mutated in greater than 10% of tumors: *EP300*, *PBRM1*, *USP28*, and *CHUK*.

*EP300* (p300) encodes a histone acetyltransferase that is a critical transcriptional co-activator of NOTCH^49^. *EP300* had frequent loss-of-function mutations, including missense mutations that clustered in the histone acetyltransferase domain (Fig. 3b). Several of these missense mutations have been functionally confirmed to eliminate histone acetyltransferase activity of the protein^50^. *EP300* has also been implicated as a tumor suppressor gene in esophageal squamous cell carcinoma^51^.

*PBRM1* encodes a subunit of the SWI/SNF chromatin remodeling complex and has been implicated as a tumor suppressor gene in a wide range of other cancers^52^. *PBRM1* had deleterious mutations occurring throughout the length of the protein (Fig. 3c). Of note, another member of the SWI/SNF chromatin remodeling complex, *ARID2*, was also implicated as a tumor suppressor gene in cutaneous squamous cell carcinoma.

*USP28* encodes a deubiquitinase that stabilizes key proteins involved in DNA repair^53^. It is required for DNA-damage-induced apoptosis mediated through the Chk2–p53–PUMA pathway^53^. *USP28* was nominated here because of its high frequency of truncating mutations (Fig. 3d).

*CHUK* encodes a protein, also known as IκB Kinase α (IKKα), that is involved in the NFκB signaling pathway. *Chuk* knockout mice are born with thickened skin, and their cutaneous keratinocytes are unable to differentiate, resulting in death shortly after birth^54^. An identical phenotype has been observed in humans with a defective *CHUK* gene^55^. These knockout mouse/human observations implicate *CHUK* as a key factor governing growth and differentiation of keratinocytes in skin. In addition, a sleeping beauty transposon screen recently demonstrated that loss-of-functional transposon insertions into the *Chuk* locus drives keratinocyte tumorigenesis in a *Pten*-sensitized mouse model^56^. Our analyses of somatic mutations were consistent with a tumor suppressive role for the *CHUK* gene, which had a high frequency of truncating mutations and somatic alterations affected both alleles in most tumors (Fig. 3e).

We also checked for genes nominated in other studies but not by our analyses. *KMT2D* was the only gene implicated in more than one of the other studies interrogated here (Supplementary Fig. 6a). The majority of *KMT2D* mutations were silent or missense mutations predicted to be benign, thus explaining why *KMT2D* was not nominated here (Supplementary Fig. 6b). However, mutations in *KMT2D* are under selection in normal skin^57^, and loss of *Kmt2d* has been functionally linked to tumor suppressive phenotypes in mouse epithelium^58^. Future studies may reveal more compelling evidence of selection in cutaneous squamous cell carcinoma.

### Recurrent pathways disrupted in cutaneous squamous cell carcinoma

**Fig. 4:**
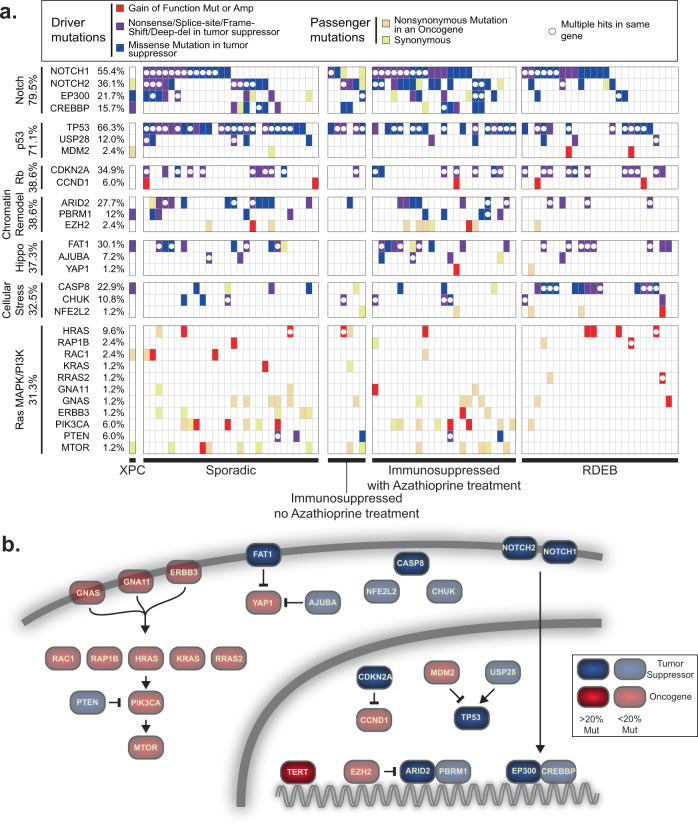
The landscape of driver mutations in cutaneous squamous cell carcinoma. **a** Tiling plot of the genetic alterations (rows) in each tumor (columns). Genes and tumors are further organized into pathways and clinical subtypes. The percentages of samples harboring pathogenic alterations are indicated. Mut mutation, Amp amplification, XPC xeroderma pigmentosum, RDEB recessive dystrophic epidermolysis bullosa. **b** Pathways affected. *TERT* was not implicated in this study, which focused on coding mutations, but is included here because it is known to have promoter mutations, as described.

The individual genes, nominated here, encode proteins that participate in a core set of signaling pathways perturbed in cutaneous squamous cell carcinoma (Fig. 4). Mutations in genes encoding proteins involved in the NOTCH and p53 pathways were ubiquitous in cutaneous squamous cell carcinomas. The NOTCH pathway had loss-of-function mutations occurring in 80% of tumors, and the p53 pathway had loss-of-function mutations occurring in 71% of tumors. Mutations in these pathways appear to be defining features of cutaneous squamous cell carcinoma.

Other pathways were recurrently disrupted, albeit to a lesser extent. Mutations that disrupt cell-cycle-checkpoint control occurred in 39% of tumors, primarily affecting the *CDKN2A* gene. Mutations that disrupt the SWI/SNF chromatin remodeling complex occurred in 38% of tumors. Mutations that activate the Hippo pathway occurred in 37% of tumors. We broadly grouped together *CASP8*, *CHUK*, and *NFE2L2*, which were collectively mutated in 33% of tumors. These genes mediate cellular responses to stress, such as inflammation and oxidative stress. More work will be needed to determine whether and how these genes are related. Finally, mutations that activate the mitogen activated protein kinase (MAPK) and/or phosphoInositide 3-kinase (PI3K) pathways occurred in 31% of tumors.

We next interrogated whether mutations affecting specific genes, pathways, or tumor subtypes overlapped more or less than would be expected by chance. Cutaneous squamous cell carcinomas from patients with recessive dystrophic epidermolysis bullosa (RDEB) were enriched with mutations affecting *CASP8* (Fig. 4a and Supplementary Data 3). CASP8 mediates cellular apoptosis in response to inflammatory cytokines^59,60^. Skin from patients with RDEB is chronically blistering and inflamed, likely explaining the selective pressure to accumulate *CASP8* mutations in this subtype of cutaneous squamous cell carcinoma. Other comparisons did not reach statistical significance after accounting for multiple hypothesis testing (see Supplementary Data 3 for a complete list of comparisons).

Most of the studies in this meta-analysis were exome-sequencing studies, prohibiting us from analyzing mutations in non-coding portions of the genome. *TERT* promoter mutations are common in many cancer subtypes, and while we were unable to investigate the locus, other studies have reported a high frequency of *TERT* promoter mutations in cutaneous squamous cell carcinoma^61^, prompting us to include *TERT* among our final list of cancer genes (Fig. 4b). Future studies will be needed to more systematically interrogate the role of non-coding mutations in cutaneous squamous cell carcinoma.

## Discussion

In this meta-analysis of exome-sequencing data, we analyzed the largest cohort of cutaneous squamous cell carcinomas to date, upheld rigorous quality control standards for sample inclusion, and utilized state-of-the-art cancer gene discovery algorithms to nominate cancer genes. In total, we nominated 30 cancer genes (Fig. 4b), known to operate in a core set of signaling pathways, that were perturbed in cutaneous squamous cell carcinoma. Our study suggests new cancer genes and helps clarify which candidates from previous studies are likely bona fide driver genes in cutaneous squamous cell carcinoma.

Future work is still needed to understand the driver genes in cutaneous squamous cell carcinoma. Cancer gene discovery studies have likely reached a saturation point for many cancers, but this is not the case for cutaneous squamous cell carcinoma. Despite the size of our meta-analysis, we could only detect cancer genes with mutations in 15% of more of tumors. We overcame this limitation, in part, by identifying genes with well-characterized hotspot mutations and/or focal copy number alterations; however, there are likely many cancer genes in cutaneous squamous cell carcinoma that have yet to be discovered. Our study also focused on the exome, prohibiting us from identifying driver mutations in non-coding regions of the genome or from identifying structural variants and viral integrations that may play a pathogenic role. Finally, as another limitation to this study, future functional and mechanistic studies will be needed to fully understand why and how the genes, discussed here, are under selection to be mutated in cutaneous squamous cell carcinoma.

Taken together, our study provides the most detailed catalog of driver genes in cutaneous squamous cell carcinoma to date, offers points of therapeutic vulnerability, and reveals critical insights into the basic biology of cutaneous squamous cell carcinoma.

## Methods

### Selection of studies

We performed a literature search to identify whole-exome or whole-genome sequencing studies of cutaneous squamous cell carcinoma that made their raw sequencing data publicly available as of September 1, 2020. The studies meeting this inclusion criteria are summarized in Table 1. The number of samples shown in Table 1 may not match the reported numbers in each study because some studies re-analyzed previously published data, or we were unable to retrieve the entirety of the raw sequencing dataset.

### Removal of 17 samples

We assessed the quality of sequencing data and removed 17 tumors from all analyses. Thirteen of these tumors had few, if any, discernible point mutations, and among the point mutations detected, their mutant allele frequencies (MAFs) were close to our detection limit. These patterns suggest poor sampling of the neoplastic cells. Two cases had less than fivefold coverage in the reference tissues, making it difficult to confidently distinguish somatic mutations from germline single nucleotide polymorphisms (SNPs). One tumor and reference pair were not properly matched, which was evident from their patterns of germline SNPs. Finally, one reference tissue had high levels of tumor contamination, prohibiting us from sensitively detecting somatic mutations.

### Calling somatic point mutations

We collected either fastq or bam files from each study. Fastq files underwent quality checks using FastQC and were subsequently aligned to the hg19 reference genome using the BWA-MEM algorithm (v0.7.13). These were further groomed and deduplicated using Genome Analysis Toolkit (v4.1.2.0) and Picard (v4.1.2.0).

Somatic point mutations were called using Mutect2 (v4.1.2.0) by comparing each tumor bam to a corresponding reference bam, thus producing an initial set of candidate somatic mutations. The variants were annotated using Funcotator (v4.1.2.0) and further filtered to remove suspected sequencing artifacts, extremely subclonal mutations, and/or mutations from unrelated clones of keratinocytes. In parallel, indels were called using Pindel (v0.2.5) and further filtered. Our filtering scripts are available here: https://github.com/darwinchangz/ShainMutectFilter. We have also deposited our mutation calls into cbioportal: https://www.cbioportal.org/study/summary?id=cscc_ucsf_2021.

To provide an overview, the script uses samtools mpileup to count the number of reference and mutant reads for each variant. Variants with low overall coverage were removed, and variants with few supporting mutant reads were also removed. Finally, we calculated tumor cellularity in each sample, and removed variants that were not predicted to be in at least 40% of tumor cells. The main reason we removed these variants is because it was difficult to distinguish whether they were from subclones within the tumor or from unrelated clones of mutant keratinocytes. Normal skin is comprised of clones of keratinocytes, many of which harbor pathogenic mutations^6,62^. We have observed that these clones commingle with adjacent skin tumors and are often unintentionally included in microdissections^63^.

### Calling heterozygous SNPs

We also identified a high-confidence set of germline heterozygous SNPs from the reference bams corresponding to each patient. Knowing these SNPs allowed us to measure allelic imbalance, thereby revealing tumor cellularity (detailed below) and corroborating copy number alterations within tumors. To identify heterozygous, germline SNPs, we called variants in the reference tissue as compared to the reference genome using FreeBayes (v1.3.1). Next, we filtered these variants to include only those that overlapped known 1000 genomes sites and which had 40–60% variant allele frequency.

### Inferring tumor cellularity

We used multiple methods, if possible, to infer the neoplastic cell content from each tumor. The methods used for each tumor are listed in Supplementary Data 1 and further described below.

“*Allelic imbalance of SNPs over deletions*”: We calculated tumor cellularity from the degree of allelic imbalance of heterozygous, germline SNPs over chromosomal arms with deletions in the tumor. This strategy assumes the deletions are fully clonal and there remains only one copy of the remaining chromosome in each tumor cell. A deletion results in a complete loss of an allele within the tumor cells. As a result, sequencing reads from the deleted allele are assumed to come from non-neoplastic cells. Tumor cellularity can therefore be calculated from ratio of reads mapping to the A and B alleles as described^63^.

“*Allelic imbalance of SNPs over copy number neutral LOH*”: Similar to the above strategy, we calculated tumor cellularity from the degree of allelic imbalance of heterozygous, germline SNPs over chromosomal arms with copy-number-neutral loss-of-heterozygosity (LOH). This strategy assumes that copy number neutral LOH is fully clonal and there are two copies of the remaining allele in each tumor cell. Copy number neutral LOH results in complete loss of an allele within the tumor cells. As a result, sequencing reads mapping to the lost allele are assumed to come from the non-neoplastic cells. Tumor cellularity can therefore be calculated from ratio of reads mapping to the A and B alleles as described^63^.

“*Modal somatic MAF*”: In addition to investigating the variant allele frequencies of heterozygous, germline SNPs, we also used the MAFs of somatic mutations. The MAF of a somatic mutation that is fully-clonal and heterozygous should be 50%, but will decrease with stromal contamination. For each tumor, we plotted a histogram of MAFs and determined the “peak” or “modal” MAF, and we doubled these values to infer tumor cellularity.

“*Median Somatic MAF*”: For a small number of tumors, the density of somatic mutations was insufficient to produce a smooth histogram. In these cases, we determined the median MAF from all somatic mutations, and we doubled this value to infer tumor cellularity. If the patient was male, we separately calculated the median MAF of somatic mutations on the sex chromosomes and incorporated these values without doubling.

### Determining statistical power to call somatic mutations (related to Supplementary Fig. 1)

To identify a somatic mutation, there must be sufficient coverage in both the reference and the tumor. Therefore, for each tumor/reference pair, we calculated the footprint for which sequencing coverage was sufficient to call somatic mutations.

In the reference, sufficient coverage is necessary to detect both alleles, thus ensuring that a variant in the tumor is a somatic mutation and not a germline SNP. We required at least sixfold coverage in the reference to call a somatic mutation. Assuming each allele is randomly sampled during sequencing, the probability of both alleles being sampled at least once with sixfold coverage is 96.9% (two-tailed binomial test). We used the Footprints software^23^ to calculate the precise number of basepairs that achieved sixfold coverage (or greater) in each reference bam and designated this value as the “call-able” footprint for each reference bam (Supplementary Data 1).

In the tumor, there needs to be sufficient coverage to detect the mutant allele. We required our somatic mutation calls to have at least four unique reads. Some mutation callers, including MuTect2, which was used in this study, will attempt to call mutations with fewer reads, but in practice, we found those calls to be of poor quality and filtered them out. We considered a site to be “call-able” if it had eightfold effective tumor coverage. “Effective tumor coverage” refers to the sequencing coverage derived from the tumor after discounting the proportion of reads from non-tumor cells. For example, if a tumor sample has 100-fold total coverage and 8% tumor cellularity, then the effective tumor coverage would be eightfold. Assuming that the alleles are randomly sampled during sequencing, their relative coverages will fit a binomial distribution and eightfold effective tumor coverage is sufficient to call a heterozygous somatic mutation 50% of the time (two-tailed binomial test). We used the Footprints software^23^ to calculate the precise number of basepairs that achieved eightfold effective tumor coverage or greater in each tumor bam and designated this value as the “call-able” footprint for each tumor bam (Supplementary Data 1).

For each tumor/reference pair, we took the minimum “call-able” footprint between the tumor and the reference and designated that value to be the “call-able” footprint for that sample. We subsequently divided the “call-able” footprint by the bait territory that was targeted to indicate the fraction of target basepairs for which we were statistically powered to recognize mutations. These numbers are reported in Supplementary Fig. 1a.

There were primarily three variables that reduced statistical power to recognize mutations: 1. Low overall coverage, 2. Low tumor cellularity, 3. Extreme variability in coverage (e.g. from GC-selection biases introduced during hybridization).

### Calling copy number alterations

Copy number alterations were inferred from the DNA- sequencing data using CNVkit^64^.

CNVkit can be run in reference or reference-free mode. We elected to run CNVkit in reference mode using the panel of normals from each study. This approach consistently produced the least noisy copy number profiles, as compared to reference-free mode or a universal reference. All other parameters were run on their default settings.

### Selecting focal somatic copy number alterations

We filtered the segmented copy number data by amplitude and level of support to create a short list of focal amplifications or deletions. Specifically, we selected amplicons with log2(tumor/reference) values above 0.9 or deep deletions with log2(tumor/reference) values below −1 We also required these segments to have at least 20 supporting probes. We then required amplicons to show evidence of allelic imbalance data to further remove technical artifacts. Finally, we manually curated and have plotted the completed list (Supplementary Figs. 3 and 4). As part of our manual inspection, when there was more than one gene within a copy number alteration, we nominated the gene we suspected to be the driver of each copy number event. For each gene that was nominated, we revisited copy number data from other samples and identified some additional examples that were previously missed with our filtering criteria—for example, we identified an extremely focal deletion of *CDKN2A* that was not supported by 20 probes. The supporting data for each copy number alteration, highlighted in this manuscript, are shown in Supplementary Figs. 3 and 4.

### Calculating tumor mutation burden and inferring mutational signature (related to Fig. 1)

When calling somatic point mutations, we only considered mutations that were estimated to be in at least 40% of tumor cells. This was helpful in comparing the mutation burdens from tumors across the different studies for which there was considerable variability in sequencing coverages. High-sequencing coverage permits the detection of subclonal mutations, which would artificially inflate the mutation burden of a tumor, compared to another with lower coverage, if subclonal mutations are counted. There were also differences in our ability to detect clonal mutations in each tumor (described in more detail in the “Determining statistical power to call somatic mutations” section). To address this issue, we divided the number of clonal mutations in each tumor by the footprint which we were statistically powered to detect mutations in each tumor.

To perform mutational signature analysis, surrounding genomic contexts were applied to single-nucleotide variants identified in each clone using the Biostrings hg19 human genome sequence package (BSgenome.Hsapiens.UCSC.hg19 v1.4.0). Variant contexts were used to assess the proportion of each clone’s mutational landscape that could be attributed to a mutagenic process using the deconstructSigs R package (v1.9.0). A set of 48 signatures recently described^28^ were analyzed. The results of these analyses are shown in the “Signature Proportion” stacked barplot of Fig. 1. In parallel, we performed a simpler analysis of dinucleotide contexts to identify cytosine to thymine transitions at the 3′ basepair of dipyrimidines or cytosine–cytosine to thymine–thymine mutations (see the “UV” column of Supplementary Data 4)—these are the classic mutation types attributed to UV radiation, and the results are shown in the “Fraction UV Mutations” stacked barplot of Fig. 1. To determine if the UV exposure in cutaneous squamous cell carcinoma is similar to exposure in normal skin, we created a 96 barplot of all mutations in our sporadic subtype (Supplementary Figure 2b). In asterisks are mutations and their trinucleotide contexts that differentiate sun-exposed and sun-shielded skin^29^.

### Nomination of driver genes

We used four cancer gene discovery programs to nominate cancer genes: MutSig^14^, LOFsigrank^35^, dN/dS^34^, and OncodriveFML^36^. dN/dS was run in covariate value mode, which combines the synonymous mutations in a gene with its epigenomic covariates to determine the background mutation rate. The other programs were run on their default settings. All genes with *q*-values of less than 0.2 are shown in Supplementary Data 2. For the purposes of this study, a gene was considered significant if its *q*-value was less than 0.05 (Fig. 2).

Next, we cross-referenced the cancerhotspots.org database to identify mutations found in our study. We included mutations with a *q*-value of less than 0.01, but relaxed this threshold for all secondary hotspots within a gene. For example, EP300 had two hotspot mutations with *q*-values of 6.9E−22 and 4.8E−07, but we also show additional hotspots in the vicinity, one with a *q*-value of 1.9E−02 and one that was a predicted hotspot using the 3D hotspot algorithm.

Finally, driver genes were curated as described in the main text to root out potential false positives.

### Removal of COL11A1 as a candidate cancer gene

*COL11A1* had a *q*-value that was on the border of significance, and one reason why *COL11A1* barely reached our significance threshold was because it had a high number of splice-site mutations. This might seem meaningful because splice-site mutations tend to be damaging, but *COL11A1* has an unusual gene structure with a large number of small exons (less than 20 amino acids), increasing the likelihood that a splice-site mutation could occur by random chance. There were very few nonsense or frameshift mutations, which are much more common in bona fide tumor suppressor genes. The cancer gene discovery algorithm which nominated *COL11A1* does not model the possibility that a splice-site mutation could occur by random chance, likely explaining why this gene escaped its filters. However, it is interesting that germline mutations in *COL7A1* cause RDEB, which in turn promotes cSCC, and it is tempting to wonder if somatic mutations in *COL11A1* could also drive cSCC. To answer this question, we compared gene expression of *COL7A1* versus *COL11A1* from the RNA-sequencing data covering cSCC and normal skin (Supplementary Fig. 5a). *COL7A1* was highly expressed in these samples, whereas *COL11A1* expression was extremely low. In addition to analyzing bulk-RNA-sequencing data, we also interrogated single-cell RNA-sequencing data from the human protein atlas project^65^. The single-cell RNA-sequencing data confirmed high expression of *COL7A1*, but not *COL11A1*, in keratinocytes from human skin (Supplementary Fig. 5b, left panels). Moreover, the human protein atlas project attempts, when feasible, to perform immunohistochemistry to validate tissue expression of each gene. Their IHC data show that *COL7A1* is highly expressed at the protein level in basal keratinocytes, whereas they could not validate any antibodies capable of detecting *COL11A1* in tissues (Supplementary Fig. 5b, right panels).

### Determining nominated genes in previous papers (related to Fig. 3 and Supplementary Fig. 6)

We sought to compare the genes nominated in our study to those nominated in previous studies. Previous studies used a variety of methodologies to nominate genes, so we outline, here, precisely how we extracted their gene lists. These genes were classified into two categories—“nominated” or “noted”. A “nominated” gene was typically highlighted in a figure, such as a tiling plot, or a gene list in the results or a table. A “noted” gene tended not reach statistical significance but was either borderline or mentioned as a potential cancer gene at some point in the manuscript.

In the Durinck paper^9^, we considered genes in the header of Table 1 as “nominated”. There was also a column in Table 1 that listed “Other known COSMIC mutations”, and we considered these to be “noted”. In the South paper^10^, we considered genes in the tiling plot of Fig. 1 to be “nominated”. The Durinck and South papers were the among the first to perform exome sequencing, and thus their gene lists were curated based on prior biological knowledge. The Cammareri paper’s “nominated” genes were those called with MutSigCV or IntOgen with a *p*-value of less than 0.05 as shown in Supplementary Data 9, and we considered genes to be “noted” from this study if they were in the tiling plots shown in Fig. 1. The “nominated” genes from the Chitsazzadeh paper were those listed as significantly mutated in Fig. 2c, which was determined by mutations being present in at least seven unique patients, or mutations which have been suggested as a driver in cutaneous squamous cell carcinoma in prior studies, or mutations with 400 or more frequency hits from the COSMIC database. The Cho paper “nominated” genes with a false discovery rate (*q*-value) of less than 0.001 using MuSiC, as shown in Fig. 1b. The Inman paper “nominated” genes featured in Fig. 3b, which were determined if a gene was called by at least two of three cancer discovery tools—the cutoff for MutSig was having a *p* value less than 0.05, and the cutoff for both OncoDrive tools was a *q* value of less than 0.05. We “noted” genes from this study that met the cutoffs for any individual tool, shown in Supplementary Data 8–10. We considered genes from the Pickering paper [47] as “nominated” if they appeared in Fig. 3 or under the heading *Copy number alterations*. These genes had a false discovery rate in MutSig of less than 0.05 or the same *q* value from at least two other statistical analyses, described in their results section. Genes not in Fig. 3, but shown in Table 4 of the Pickering paper with at least one mutation in their cohort, were considered by us as “noted”. In the Li, Y.Y. paper, we took genes to be “nominated” if they were listed in Fig. 1b—these genes had a false discovery rate (*q* value) of less than 0.1 using MutSig. The Li manuscript also “noted” other genes, which were mentioned in both Fig. 1b and Fig. 3 that did not reach statistical significance in MutSig but had copy number alterations in their cohort or roles in other cancer types. In total, these studies “nominated” 70 unique genes and “noted” 335 unique genes.

### RNA-Sequencing analysis (related to Supplementary Fig. 5)

Two of the studies analyzed in this meta-analysis had RNA-sequencing data available^5,6^. These datasets covered both normal skin (*n* = 17 samples) and cutaneous squamous cell carcinoma (*n* = 17 samples). We downloaded the raw sequencing data, aligned with STAR, and quantified gene expression with RSEM, as previously described^66^. In Supplementary Fig. 5, we show the fragment per kilobase of transcript per million reads (FPKM) values for candidate genes (see Fig. 2 for a list of candidates). FPKM values normalize for gene length and read depth, allowing the comparison of gene expression levels across genes. In Supplementary Fig. 5c, we combined RNA-sequencing reads from all 34 samples over the *KNSTRN* gene, demonstrating that the mutant hostpot is not expressed.

### Mutational overlap analysis (related to Supplementary Data 3)

We interrogated whether mutations affecting specific genes, pathways, or tumor subtypes overlapped more or less than would be expected by chance. We restricted our analyses to genes, pathways, or tumor subtypes with at least 16 mutant tumors—the minimum number that could reach statistical significance with our sample size. *P* values for individual comparisons were calculated using the Fisher’s exact test. We corrected for multiple hypothesis testing by computing false discovery rates (*q* values) using the Benjamini–Hochberg procedure. A full list of *p* and q values for each comparison is shown in Supplementary Data 3.

### Reporting summary

Further information on research design is available in the Nature Research Reporting Summary linked to this article.

## Supplementary information

Supplementary Information

Supplementary Data 1

Supplementary Data 2

Supplementary Data 3

Supplementary Data 4

Reporting Summary

## Data Availability

Raw sequencing data from previously published manuscripts were obtained from European Genome-Phenome Archive accession number EGAD00001003555 (refs. ^7,8,10^), dbGaP accession number phs000830.v1.p1 (ref. ^12^), SRA accession number SRP134188 (ref. ^5^), SRA accession number SRP265179 (ref. ^13^), or at the request of the corresponding authors (refs. ^4,6,9,11^). Mutation calls and other analyses, performed by us, are available in the supplementary tables. The somatic mutation calls have also been deposited into cbioportal (https://www.cbioportal.org/).

## Code availability

Custom scripts and program versions used to generate mutation lists are available here: (https://github.com/darwinchangz/ShainMutectFilter).
