# Supplementary material for: The landscape of driver mutations in cutaneous squamous cell carcinoma
Source: NPJ Genom Med. 2021 Jul 16;6:61. doi: 10.1038/s41525-021-00226-4 (PMC8285521; doi:10.1038/s41525-021-00226-4)
Supplement: Supplementary file 1 — Supplementary Information [file 41525_2021_226_MOESM1_ESM.pdf]

Supplementary information associated with “The landscape of driver mutations in cutaneous squamous cell carcinoma”

Darwin Chang[1,2], A. Hunter Shain[1,2]

[1] University of California San Francisco, Department of Dermatology

[2] University of California San Francisco, Helen Diller Family Comprehensive Cancer Center

Table of Contents

Page 1 – Table of Contents

Page 2 – Supplementary Data Legends

Page 3-8 – Supplementary Figures 1-6

Supplementary Data 1. A summary of samples included in this study and their sequencing metrics. We identified 105 cutaneous squamous cell carcinomas from 101 patients (4 patients had multiple tumors) for which sequencing data was publicly available. This table summarizes available information pertaining to these patients and their tumors as well as relevant sequencing metrics. Note that some tumors had whole genome sequencing, but we restricted our analyses to the exome. Methods to infer tumor cellularity are explained in more detail under *Inferring Tumor Cellularity* in the methods section. RDEB = Recessive Dystrophic Epidermolysis Bullosa. XPC = Xeroderma Pigmentosum.

Supplementary Data 2. Genes nominated to be under positive selection in cutaneous squamous cell carcinoma. All genes nominated by each of the four cancer gene discovery tools with a q-value below 0.2 are shown. For the purposes of this study, we considered a gene to significant if it had a q-value of less than 0.05. In the first tab, we show genes nominated from an analysis of all tumors passing quality control, and in the subsequent tabs, we show the genes nominated from analysis of clinically distinct types of cutaneous squamous cell carcinoma.

Supplementary Data 3. Genes, pathways, and tumor subtypes with significantly overlapping (or non-overlapping) mutations. P-values reflect the results of a two-tailed Fisher exact test, and q-values account for multiple hypothesis testing using the Benjamini-Hochberg procedure.

Supplementary Data 4. Somatic mutations in cutaneous squamous cell carcinoma. Somatic mutations that passed filtering were annotated using Funcotator and shown here. These mutations are also available on cbioportal.

Supplementary Figure 1.

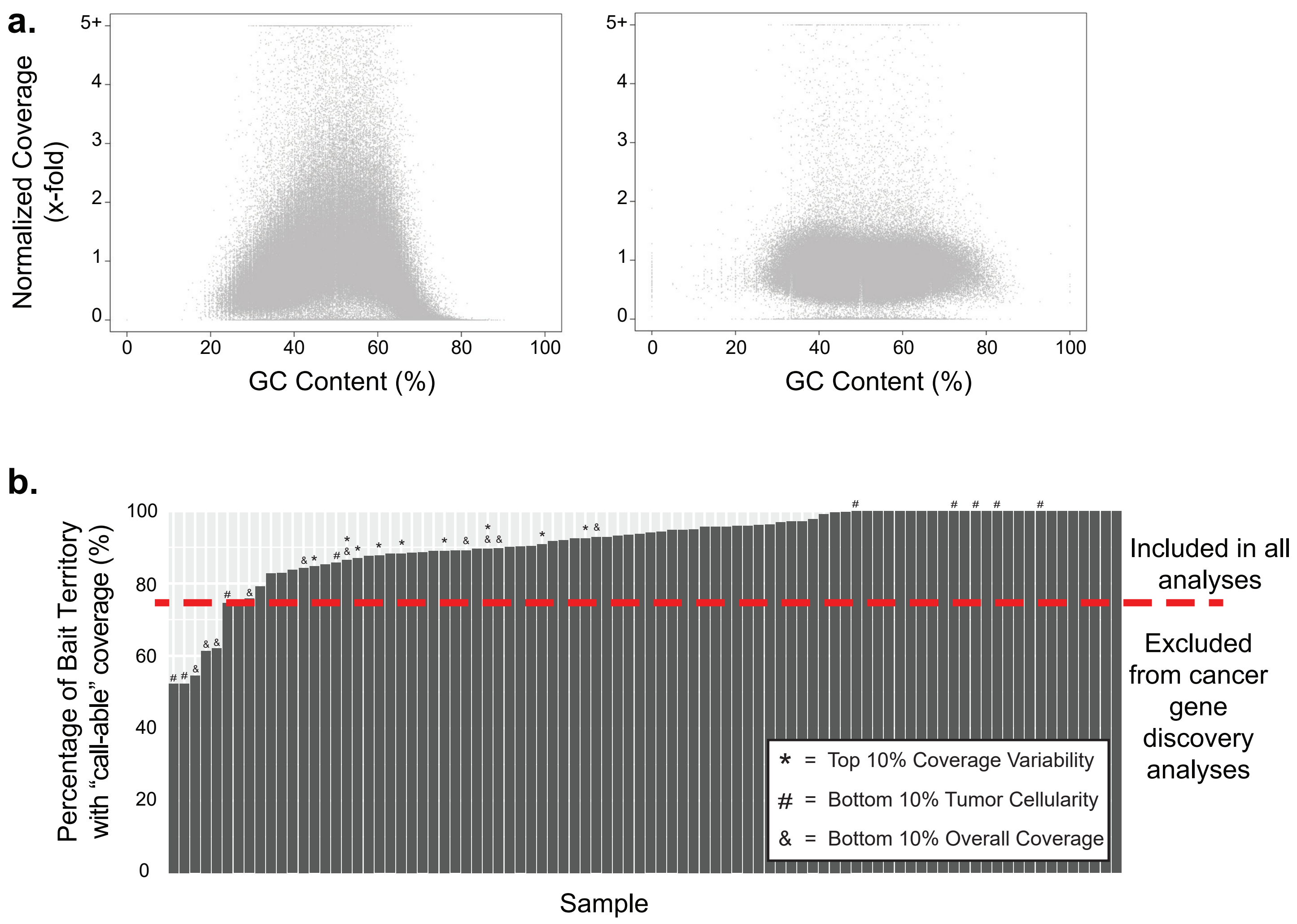

Supplementary Figure 1. Ability to detect mutations in each tumor varies substantially. **a.** Some tumors had high overall coverage but extreme variability in coverage, primarily linked to GC content, thus diminishing our ability to detect mutations within large portions of the bait territory. An example of a tumor in the top 10% of coverage variability (left panel) and a tumor in the bottom 10% of coverage variability (right panel). Each datapoint corresponds to a bait interval, stratified by its GC content and sample-normalized coverage. **b.** The percentage of base pairs within the target bait territory with sufficient coverage to call a mutation. Tumors with low overall sequencing coverage, extreme variability in sequencing coverage, and/or low tumor cellularity tended to have a lower proportion of "call-able" mutations. Tumors with greater than 75% "call-able" mutations were included in all analyses, whereas those with fewer were only included in certain analyses, as described.

Supplementary Figure 2.

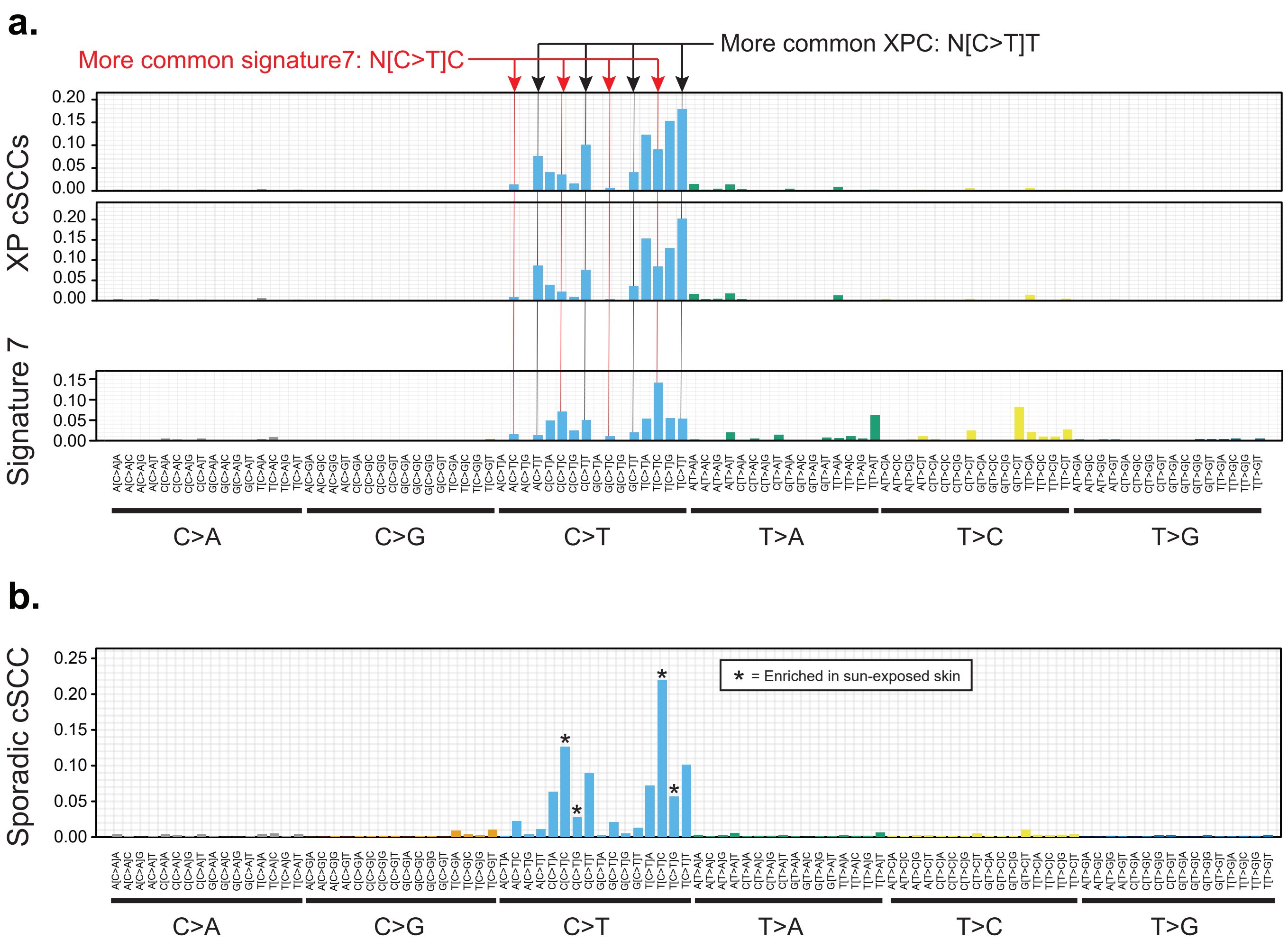

Supplementary Figure 2. Somatic mutations in Xeroderma Pigmentosum tumors occur in a distinct tri-nucleotide context. a. The tri-nucleotide context of mutations in two XPC-/- tumors as compared to signature 7. C>T transitions at dipyrimidines were common in both, however the basepair downstream (three prime) to the mutation site differed with thymines more common in XPC-/- tumors and cytosines more common in signature 7. **b.** The tri-nucleotide context of mutations in sporadic cSCCs. Asterisks denote mutations enriched from a comparison of sun-exposed skin to non-exposed skin from Wei/Christensen et al.[29].

Supplementary Figure 3.

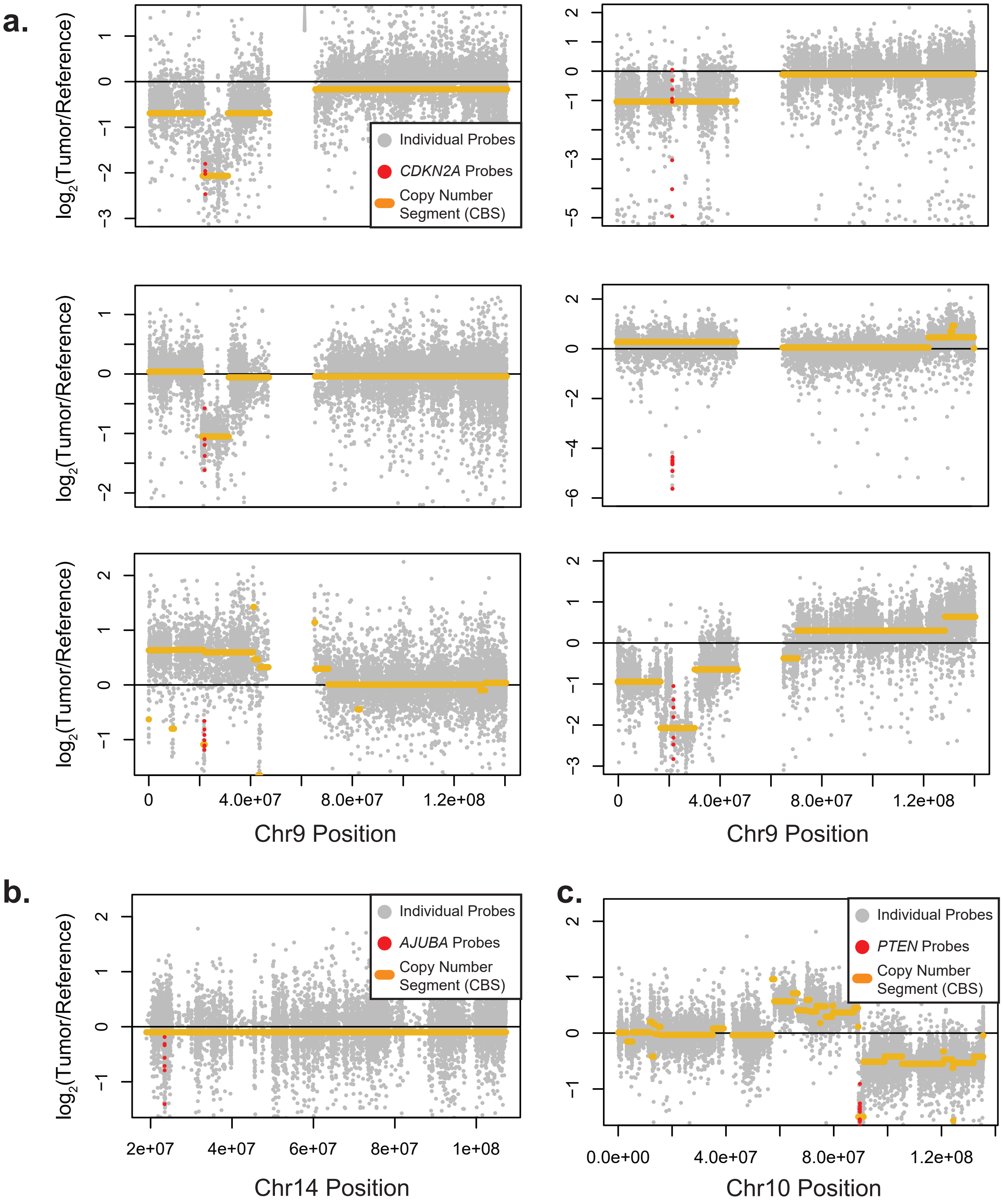

Supplementary Figure 3. Focal deletions affecting *CDKN2A*, *AJUBA*, and *PTEN*. Copy number over individual genomic positions (data points) with copy number segments (yellow lines) overlaid. Colored data points correspond to probes covering *CDKN2A* (panel **a**), *AJUBA* (panel **b**), and *PTEN* (panel **c**).

Supplementary Figure 4.

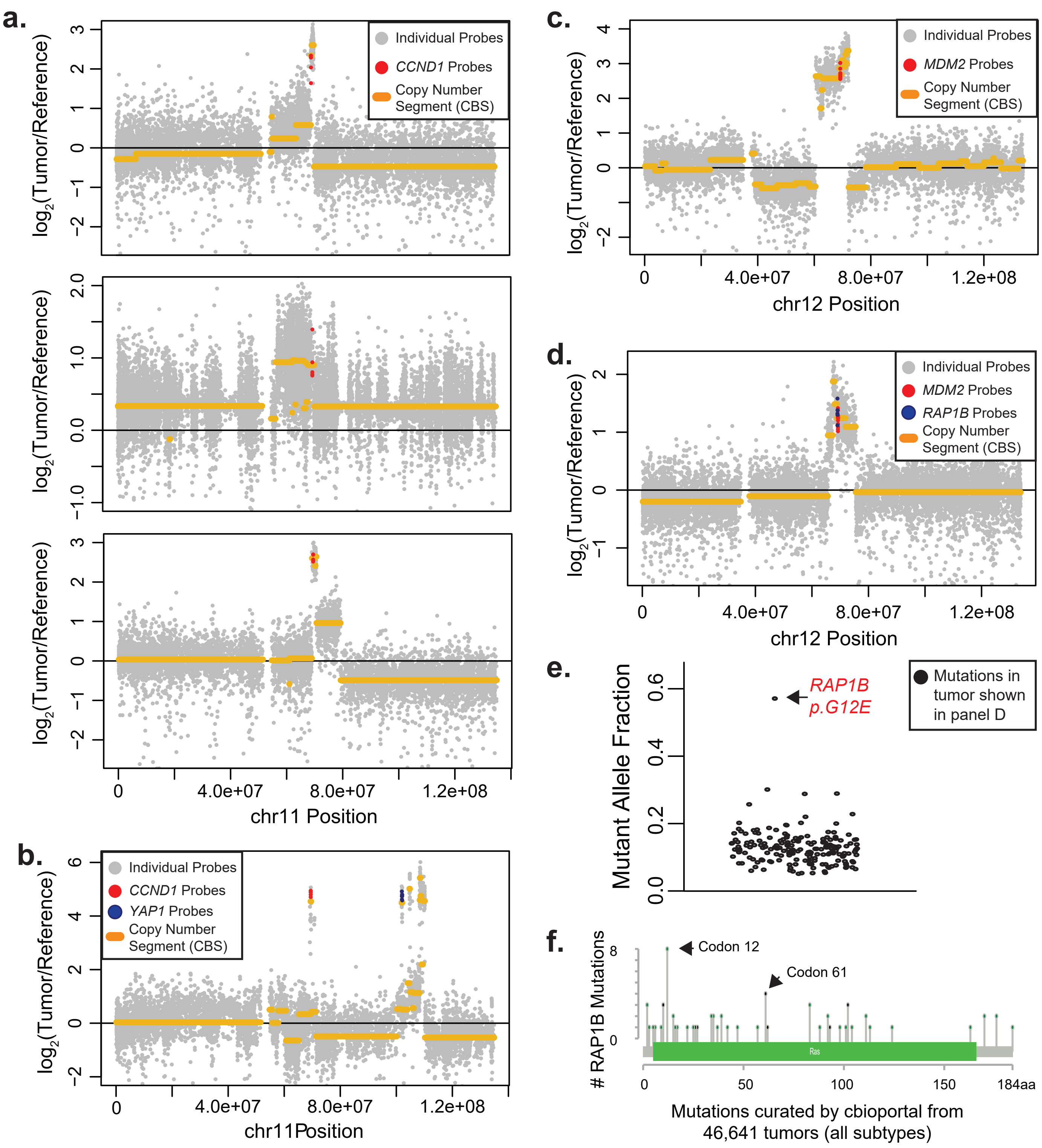

Supplementary Figure 4. Focal amplification affecting *CCND1*, *YAP1*, *MDM2*, and/or *RAP1B* in six tumors. Copy number over individual genomic positions (data points) with copy number segments (yellow lines) overlaid. Colored data points correspond to probes covering *CCND1* (panels **a** and **b**), *YAP1* (panel **b**), *MDM2* (panels **c** and **d**), and *RAP1B* (panel **d**). Note that amplifications converge upon the noted genes across these six tumors. The tumor with amplification of *RAP1B* (shown in panel **d**) also had a point mutation affecting the 12th codon of *RAP1B* (panel **e**). The mutant allele frequency of this point mutation was higher than all other mutations in that tumor, consistent with the mutation affecting the amplified allele. RAP1B is a ras-like protein with recurrent mutations affecting codons 12 and 61 across cancer (panel **f**), analogous to mutations known to activate other ras-like proteins.

Supplementary Figure 5.

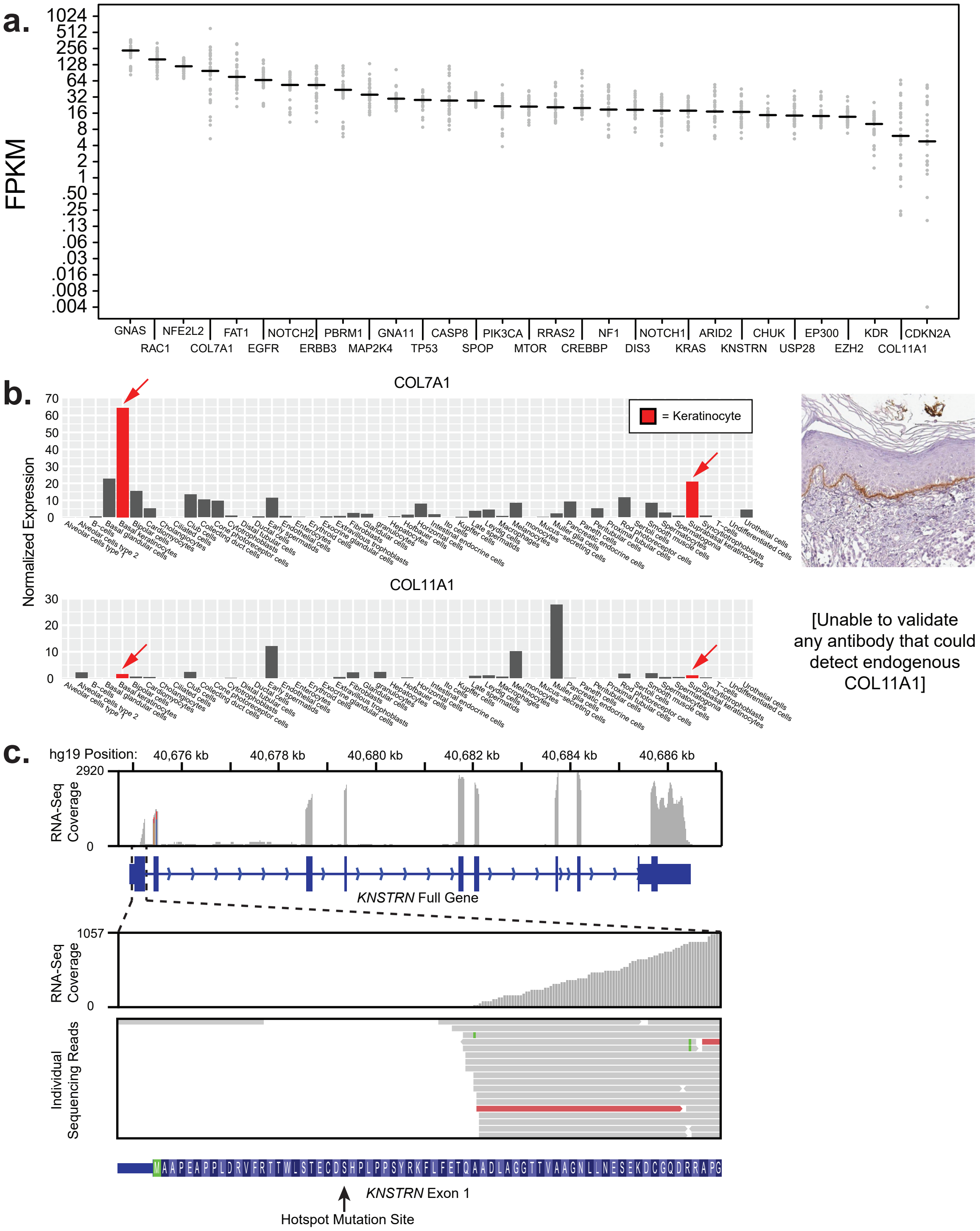

Supplementary Figure 5. Gene expression of candidate genes. a. The Fragments Per Kilobase of transcript per Million reads (FPKM) values -- a measurement that normalizes for both gene length and sequencing depth in RNA-sequencing data -- of candidate genes. RNA-sequencing data derived from both normal skin samples (n=17 samples) and cutaneous squamous cell carcinomas (n=17 tumors). This data was used to flag potential false positive candidates based on their poor expression in normal and neoplastic keratinocytes. **b.** Normalized expression of *COL7A1* and *COL11A1* in various cell types from the human protein atlas project (keratinocytes are highlighted in red). Protein levels in human skin from the human protein atlas are shown on the right. **c.** Transcription of *KNSTRN* begins downstream of the hotspot mutation site. RNA-sequencing data was aggregated from normal skin (n=17) and cutaneous squamous cell carcinoma (n=17). Top panel -- RNA-sequencing coverage over the *KNSTRN* gene. Coverage was approximately 2,000 to 3,000-fold over most exons with the exception of exon1. Bottom panel -- A zoomed inset of RNA-sequencing coverage over exon1. While sequencing coverage surpassed 1,000-fold at the three-prime junction of exon1, there were no reads spanning the hotspot mutation site from the any of the 34 samples, in-line with our prediction that the mutation is non-coding.

Supplementary Figure 6.

a.

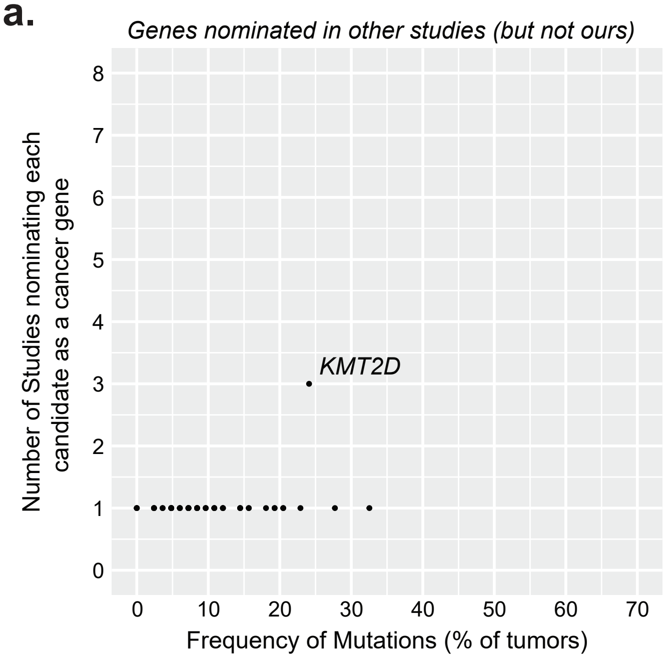

b.

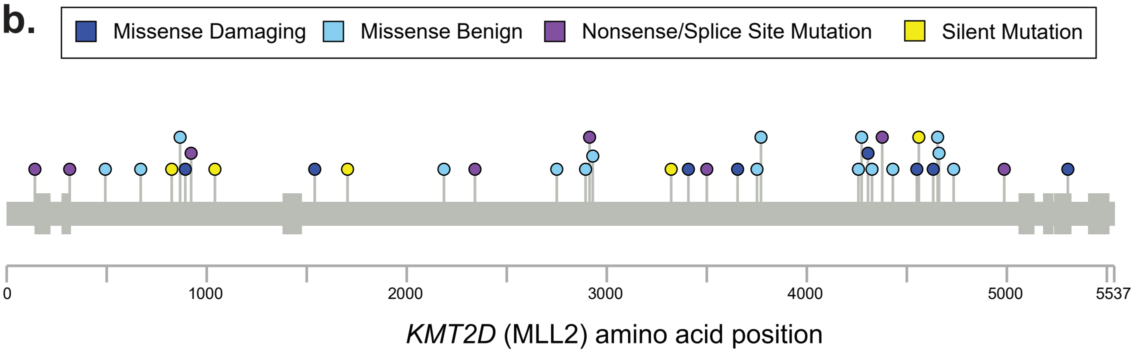

Supplementary Figure 6. *KMT2D* has a benign spectrum of mutations. a. Genes nominated by other studies, but not our study, are stratified by their mutation frequency (x-axis) and how often they were nominated in 8 previous studies (y-axis) that catalogued drivers of cutaneous squamous cell carcinoma. *KMT2D* was the only gene recurrently implicated in other studies but not ours. **b.** Lollipop diagram portrays the spectrum of mutations. *KMT2D* was not nominated by cancer gene discovery algorithms because of its high frequency of silent mutations and missense mutations predicted to be benign.
